# Use of near-infrared reflectance spectroscopy for the rapid determination of the digestible energy and metabolizable energy content of corn fed to growing pigs

**DOI:** 10.1186/s40104-016-0105-9

**Published:** 2016-08-04

**Authors:** Juntao Li, Quanfeng Li, Defa Li, Yiqiang Chen, Xiaoxiao Wang, Wunjun Yang, Liying Zhang

**Affiliations:** State Key Lab of Animal Nutrition, College of Animal Science & Technology, China Agricultural University, Beijing, 100193 China

**Keywords:** Corn, Digestible energy, Growing pigs, Metabolizable energy, Near-infrared reflectance spectroscopy

## Abstract

**Background:**

The ability of near-infrared reflectance spectroscopy (NIRS) to determine the digestible energy (DE) and metabolizable energy (ME) content of corn fed to growing pigs was tested. One hundred and seventeen corn samples, comprising different planting regions and varieties were collected from all over China in a three-year period. The samples were randomly split into a calibration set (*n* = 88) and a validation set (*n* = 29). The actual and calculated DE and ME content of the corn samples was determined by digestion-metabolism experiments and the prediction equations of Noblet and Perez (J Anim Sci. 71:3389–98,1993). The samples were then subjected to NIRS scanning and calibrations were performed by the modified partial least square (MPLS) regression method based on 77 different spectral pre-treatments. The NIRS equations based on the actually determined and calculated DE and ME were built separately and then validated using validation samples.

**Results:**

The NIRS equations obtained from actually determined DE, the coefficient of determination for calibration (RSQ_cal_), cross-validation (R^2^_CV_), and validation (RSQ_v_) were 0.89, 0.87 and 0.86, and these values for determined ME were 0.87, 0.86 and 0.86. For the NIRS equations built from calculated DE, the RSQ_cal_, R^2^_CV_, and RSQ_v_ values were 0.88, 0.85 and 0.84, and these values for calculated ME were 0.86, 0.84 and 0.82. Except for the equation based on calculated ME (RPD_v_ = 2.38, < 2.50), the other three equations built from actually determined energy and calculated DE produced good prediction performance (RPD_v_ ranging from 2.53 to 2.69, > 2.50) when applied to validation samples.

**Conclusion:**

These results indicate that NIRS can be used as a quantitative method for the rapid determination of the available energy in corn fed to growing pigs, and the NIRS equations based on the actually determined energy produced better predictive performance than those built from calculated energy values.

## Background

The cost of feed usually represents more than 70 % of the total cost of pork production and feed energy generally represents the single largest component of this expense [1]. Corn plays a key role in providing energy in typical Chinese wine diets. In the production of swine feeds in China, formulas are typically made based on the digestible energy (DE) and metabolizable energy (ME) values recommended by NRC [2] which only provides the average DE and ME value (just about DE and ME value) for corn. However, because of the use of different corn varieties, planting regions, storage conditions and processing methods, the nutrient levels vary greatly among different sources of corn. Zhao et al. [3] have analyzed the nutritional values of 30 corn samples collected from China, the results indicated that the nutrients varied largely between different samples, ranging from 8.5 to 11.9 % for crude protein (CP), 2.3 to 5.3 % for ether extract (EE), 0.8 to 1.5 % for ash, 1.1 to 3.7 % for crude fiber (CF), 6.0 to 21.8 % for neutral detergent fiber (NDF) and 1.8 to 6.8 % for acid detergent fiber (ADF). These differences will typically cause large variations in the DE and ME content of corn when fed to growing pigs [4], and thus will have economic implications for swine producers. Therefore, in order to achieve accurate feed formulation, and decrease the cost of pork production, it is important to precisely determine the actual DE and ME content of corn before its use.

At present, the evaluation of DE and ME of feed ingredients is mainly made through traditional digestion-metabolism experiments, which are time consuming, labor intensive, expensive and can potentially pollute the environment [5]. Therefore, it is essential to establish a rapid and accurate method to measure the energy value of feed ingredients. Based on the analysis of chemical components, several equations have been proposed to estimate the energy values of complete diets [6, 7] and feed ingredients, including corn [8], barley [9], corn co-products [10, 11], and corn gluten meals [12] fed to pigs, but this approach is limited by its lack of speed and poor repeatability.

As a rapid, non-destructive and relatively inexpensive technology, near-infrared reflectance spectroscopy (NIRS) has been successfully applied in the feed industry to predict the chemical composition of corn [13–15]. Some studies have also investigated the possibility of predicting the DE and ME content in barley [4, 16] and wheat [17, 18] by NIRS, but the results obtained were not satisfying due to the low number of samples used and relatively small inter-sample variability. In addition, to the best of our knowledge, there has been no report about the rapid prediction of swine DE and ME content in corn by NIRS. In this study, to improve the performance of this technology, the number of collected corn samples was increased and the inter-sample variability was enlarged by picking corn samples from different planting regions and varieties. Furthermore, a comparison was made between the NIRS equations derived from reference data determined by metabolism experiments and the prediction equations of Noblet and Perez [6].

## Methods

### Sample preparation

Between 2009 and 2011, a total of 117 corn samples from different planting regions and varieties were collected from all over China. Eighty eight corn samples were randomly chosen as a calibration set and the remaining 29 samples were used as a validation set. For the development of NIRS calibrations, corn samples were ground in a Universal High-speed Grinder FW-100 (Ever Bright Medical Instrument Co., company, Beijing, China) through a 0.42 mm screen. Samples were stored at -18 °C and brought to room temperature (24 °C) prior to chemical analysis and NIRS scanning.

### Reference data analysis

Samples were analyzed using the methods of AOAC International [19]. Analysis were conducted for moisture (AOAC method 930.15), crude protein (CP; AOAC method 999.03), ash (AOAC method 975.03) and ether extract (EE, AOAC method 2003.06). Neutral detergent fiber (NDF) and acid detergent fiber (ADF) were determined using filter bags and fiber analyzer equipment (Fiber Analyzer, Ankom Technology, Macedon, NY) following a modification of the procedure of van Soest et al. [20]. The concentration of NDF was analyzed using heat stable α- amylase and sodium sulphite without correction for insoluble ash. The ADF fraction was analyzed in a separate sample. The DE and ME contents of corn samples were determined by both digestion-metabolism experiments and the prediction equations of Noblet and Perez [6].

The Animal Welfare Committee of China Agricultural University (Beijing, China) approved the animal care protocol used for the digestion-metabolism experiments. One hundred and seventeen diets were formulated to include one specific corn sample (96.8 %), dicalcium phosphate (1.7 %), limestone (0.6 %), salt (0.3 %), mineral and vitamin premix (0.5 %) and antioxidant (0.1 %). Corn was considered to be the only source of energy in the diet, assuming that the contribution of energy from vitamin and mineral premixes was negligible. Vitamins and minerals were supplied at levels exceeding the requirements of 20 to 50 kg growing pigs recommended by NRC [21].

The whole experiment consisted of six digestibility trials and was conducted from October 2011 to May 2012 under similar experimental conditions. There are 10 metabolism rooms, and each room has 12 metabolism cages. Twenty diets were measured for each of the first five trials, and 17 diets were measured for the sixth trial. A total of 702 growing crossbred barrows [(Yorkshire × Landrace) × Duroc] with similar genetic background and initial weight of 35 ± 1.2 kg were used according to a completely randomized design, and each diet was tested on six pigs.

Pigs were housed individually in stainless steel metabolism cages (1.4 m × 0.45 m × 0.6 m) in an environmentally controlled room (22 ± 2 °C). The daily feed allowance was equivalent to 4 % of body weight at the beginning of each period [22]. It was divided into two equal parts and fed at 0800 and 1700 h in mash form. Water was available *ad libitum* through a drinking nipple. Pigs were fed experimental diets for 14 d, including 7 d for adaptation and 5 d for fecal and urine collection. The fecal marker (10 g/kg) were included in the morning meal on d 8 (chromic oxide) and in the morning meal on d 13 (ferric oxide), and fecal collections were initiated when chromic oxide appeared in the feces and ceased when ferric oxide appeared [22]. Urine collections were started on d 8 at 1700 h and ceased on d 11 at 1700 h. The collection and sample preparation for feces and urine were conducted according to the methods described by Song et al. [23]. The gross energy (GE) in corn samples, diets, feces, and urine samples was analyzed via Adiabatic Oxygen Bomb Calorimeter (Parr Instruments, Moline, IL). Two pieces of well-folded filter paper with known quality were placed in the crucible which was provided by the Adiabatic Oxygen Bomb Calorimeter (Parr Instruments, Moline, IL), and then 4 mL of each urine sample was added to the filter paper. After that, the filter paper with crucible were put into a vacuum dryer for drying at 60 °C. The dried filter paper with crucible were placed in the Adiabatic Oxygen Bomb Calorimeter (Parr Instruments, Moline, IL) for the analysis of total gross energy. At last, the combustion heat of the filter paper was determined and deducted from the total gross energy. According to the method above, the gross energy of urine samples were analyzed. The DE and ME contents of corn were calculated by the direct method [22].

DE and ME (dry matter basis) of corn samples fed to growing pigs were also calculated using the following equations of Noblet and Perez [6]:

$$ \mathrm{D}\mathrm{E}\ \left(\mathrm{M}\mathrm{J}/\mathrm{kg}\right)\kern0.5em =\kern0.5em \left(4,168\kern0.5em \hbox{--} \kern0.5em 9.1\kern0.5em \times \kern0.5em \mathrm{Ash}\kern0.5em +\kern0.5em 1.9\kern0.5em \times \kern0.5em \mathrm{C}\mathrm{P}\kern0.5em +\kern0.5em 3.9\kern0.5em \times \kern0.5em \mathrm{E}\mathrm{E}\kern0.5em \hbox{--} \kern0.5em 3.6\kern0.5em \times \kern0.5em \mathrm{N}\mathrm{D}\mathrm{F}\right)\kern0.5em \times \kern0.5em 4.18\ /1,000 $$

$$ \mathrm{ME}\kern0.5em \left(\mathrm{M}\mathrm{J}/\mathrm{kg}\right)\kern0.5em =\kern0.5em \mathrm{D}\mathrm{E} \times \left(1.003\kern0.5em \hbox{--} \kern0.5em 0.00021\kern0.5em \times \kern0.5em \mathrm{C}\mathrm{P}\right) $$

The standard error of laboratory (SEL) was calculated according to the method of Kovalenko et al. [24]. The relative standard error of laboratory (RSEL) was calculated as SEL/mean × 100 %.

### NIRS spectra collection

Spectral data collection, processing and calibration were conducted with the chemometrics software WinISI II Ver. 1.50 (Infrasoft International, Port Matida, PA). Spectral measurements were performed using a FOSS NIRSystem 6500 Spectrophotometer (FOSS NIRSystems Inc., Silver Springs, MD). Samples were placed in a 1/4 rectangular cup (5.7  cm× 4.6 cm) and then scanned in the diffused-reflectance mode. Each spectrum represented the average of 32 scans and was recorded as the logarithm of the reciprocal of reflectance (log (1/R)). Each sample was measured in two independent subsamples and the average spectrum was used for chemometric analysis [25]. Data were stored at every 2 nm interval in the wavelength range from 400 to 2,498 nm [25].

### Calibration and validation process

All calibration equations were developed using the modified partial least square (MPLS) regression method using the calibration set (*n* = 88) [26]. Seventy seven different spectral pre-treatments including 7 scatter correction methods combined with 11 mathematical treatments (0,0,1,1; 1,4,4,1; 2,4,4,1; 1,8,8,1; 2,8,8,1; 1,10,10,1; 2,10,10,1; 1,12,12,1; 2,12,12,1; 1,16,16,1; 2,16,16,1) were used. They include a mathematical treatment that uses the raw spectra, or their first or second derivatives (to remove background differences whiles enhancing spectral differences); combined with gap sizes in data points over which the derivative is calculated; and a smoothing algorithm that reduces random noise in the spectral data. For example in 2,4,4,1, the first number indicates the order of derivative function (two is the second derivative of log (1/R)); the second number is the gap (length in nm); the third number represents the number of data points (segment length) used in the first smoothing and the fourth number is the number of data points in the second smoothing which is normally set at 1 for no second smoothing. The scatter correction methods included Original Data (None), Standard Normal Variate plus Detrend correction (SNVD), Standard Normal Variate (SNV), Detrend correction (Detrend), Standard Multiplicative Scatter Correction (SMSC), Weighted Multiplicative Scatter Correction (WMSC) and Inverse Multiplicative Scatter Correction (IMSC).

Cross-validation was used to select the optimal number of partial least square factors and to avoid overfitting [27]. The calibration set was divided into 6 cross-validation groups. The optimal number of factors was considered as that which produced the minimum standard error of cross-validation (SECV). The calibration was developed using a maximum of two passes for outlier elimination. Outliers were defined as *H* outliers (global *H* ≥ 10, spectral outliers) [28] and *T* outliers (*T* > 2.5, samples which did not fit the calibration model).

Calibration models were assessed by statistical parameters including the coefficient of determination for calibration (RSQ_cal_), the standard error of calibration (SEC), the coefficient of determination for cross-validation (R^2^_CV_), and the standard error of cross-validation (SECV). Optimum calibrations equations were obtained with the highest RSQ_cal_ or R^2^_CV_ and the lowest SEC or SECV values. The ratio of standard deviation (SD) of the original data to the SECV (ratio of prediction to deviation, RPD) [29] was also used to evaluate calibration performance.

Validation was performed using the validation set (*n* = 29). The coefficient of determination for validation (RSQ_v_) and standard error of prediction (SEP) invalidation were used to test whether the equations obtained had good predictive performance. RPD value was expressed as RPD_v_ (SD/SEP) for validation. NIRS equations with RPD (RPD_cv_ and RPD_v_) values greater than 2.50 can be successfully applied to routine analysis [30].

## Results

### Variability in energy content

Table 1 shows the chemical composition and the DE and ME content of the corn fed to growing pigs. As for the entire set of samples, the concentration of CP, EE, ash, ADF and NDF in DM ranged from 7.80 to 11.00 %, 2.09 to 4.97 %, 0.83 to 1.82 %, 1.73 to 3.69 % and 6.40 to 19.37 %, respectively. The standard error of laboratory (SEL) and relative standard error of laboratory (RSEL) values for these chemical components ranged from 0.015 to 0.228 % and 0.62 to 2.29 %, respectively. The actual DE and ME values determined by digestion-metabolism experiments (DE_D_ and ME_D_) varied from 14.99 to 17.50 MJ/kg DM and 14.42 to 17.05 MJ/kg DM, respectively, while these values calculated according to the equations of Noblet and Perez [6] (DE_c_ and ME_c_) varied from 14.74 to 17.72 MJ/kg DM and 14.43 to 17.42 MJ/kg DM, respectively. The entire set, calibration set and validation set for each chemical component and energy fraction had similar sample distributions with similar mean values, standard deviations (SD) and coefficients of variation (CV).

**Table 1:** Chemical composition and digestible energy (DE) and metabolizable energy (ME) content of corn fed to growing pigs determined directly or by prediction equations (MJ/kg, dry matter basis)

	Entire set (*n* = 117)							Calibration set (*n* = 88)					Validation set (*n* = 29)				
Items^e^	Min	Max	Mean	SD^a^	CV^b^	SEL^c^	RSEL^d^	Min	Max	Mean	SD^a^	CV^b^	Min	Max	Mean	SD^a^	CV^b^
Moisture, %	10.90	14.00	12.63	0.892	7.06	0.129	1.02	10.90	14.00	12.61	0.930	7.37	10.94	13.96	12.69	0.881	6.94
CP, %	7.80	11.00	9.41	0.718	7.63	0.057	0.62	7.80	11.00	9.42	0.709	7.53	7.85	10.95	9.39	0.732	7.80
EE, %	2.09	4.97	3.87	0.580	14.99	0.025	0.64	2.09	4.97	3.88	0.592	15.25	2.13	4.91	3.85	0.572	14.86
Ash, %	0.83	1.82	1.41	0.257	18.21	0.015	1.06	0.83	1.82	1.40	0.258	18.45	0.85	1.78	1.43	0.253	17.67
ADF, %	1.73	3.69	2.33	0.443	19.02	0.053	2.29	1.73	3.69	2.31	0.459	19.89	1.81	3.59	2.38	0.444	18.64
NDF, %	6.40	19.37	12.02	3.083	25.65	0.228	1.91	6.40	19.37	11.98	3.165	26.42	7.23	19.19	12.11	3.039	25.10
DE_D_^f^	14.99	17.50	16.37	0.751	4.59	-	-	14.99	17.50	16.39	0.757	4.62	15.02	17.48	16.31	0.741	4.54
ME_D_^f^	14.42	17.05	15.96	0.734	4.60	-	-	14.42	17.05	15.97	0.740	4.64	14.49	17.02	15.93	0.730	4.58
DE_C_^g^	14.74	17.72	16.42	0.718	4.37	-	-	14.74	17.72	16.43	0.725	4.41	14.81	17.69	16.38	0.708	4.32
ME_C_^g^	14.43	17.42	16.16	0.697	4.31	-	-	14.43	17.42	16.18	0.709	4.38	14.61	17.35	16.10	0.676	4.20

^a^Standard deviation

^b^Coefficient of variation

^c^Standard error of laboratory

^d^Relative standard error of laboratory

^e^ *CP* crude protein, *EE* ether extract, *ADF* acid detergent fiber, *NDF* neutral detergent fiber

^f^DE_D_ and ME_D_ values determined by digestion-metabolism experiments using growing pigs

^g^DE_C_ and ME_C_ values calculated according to the equations of Noblet and Perez [6]

### Spectral pre-treatment

**Fig. 1:**
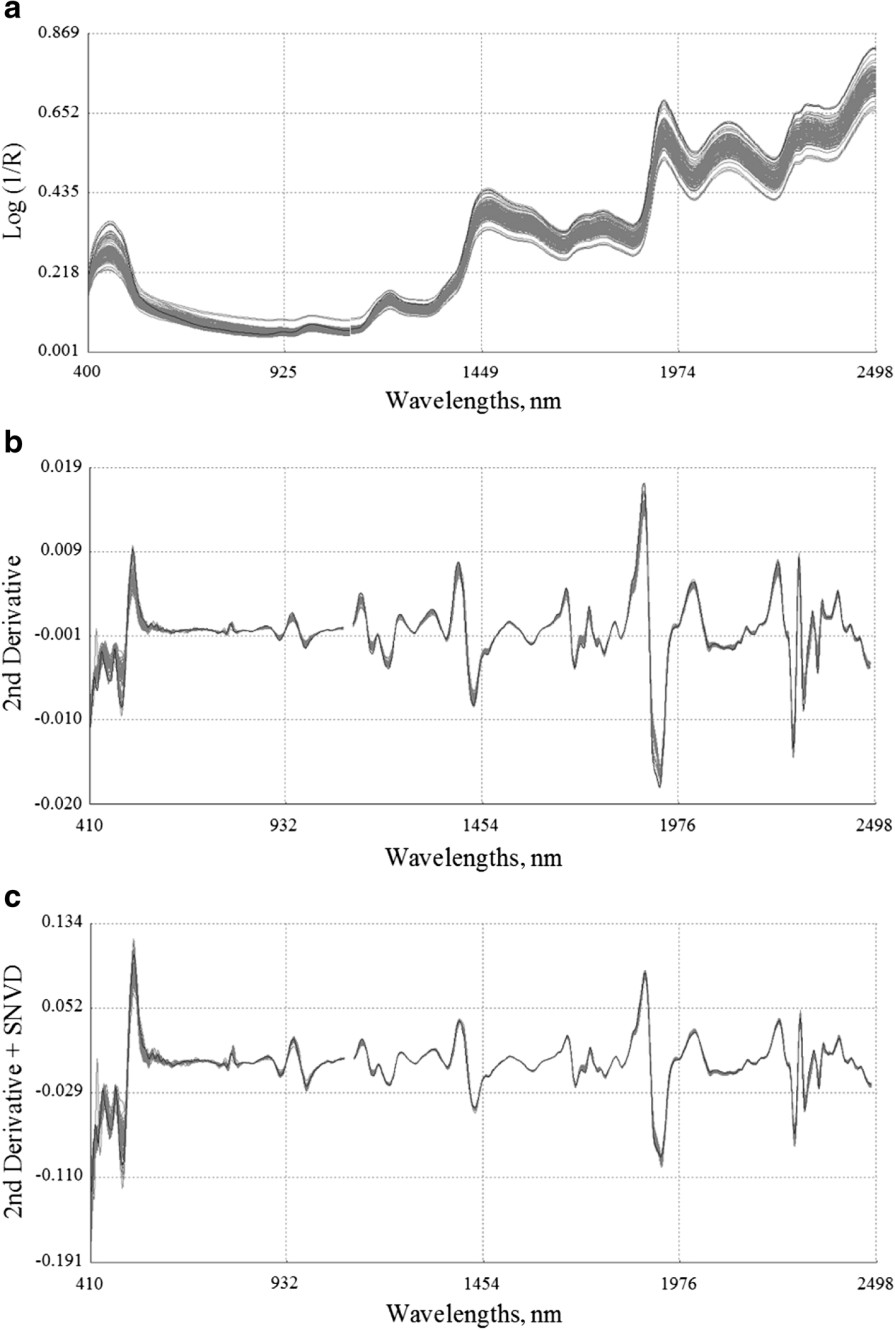
Reflectance spectra of corn (*n* = 117) in different spectral pre-treatments. (**a**) Raw spectra; (**b**): Derivative 2,4,4,1; (**c**): Derivative 2,4,4,1 + Standard normal variate plus detrend correction (SNVD)

Figure 1 shows the reflectance spectra of corn samples using different spectral pre-treatments. The original NIR spectra of all the corn samples used in this study are shown in Fig. 1 (a). Besides broad peaks and considerable baseline shifts, parallel shifts between the spectra caused by scattering of samples were also observed [31]. In order to resolve these problems, a number of mathematical treatments combined with different scatter correction algorithms were tested on the spectra. As shown in Fig. 1 (b), after being treated with the 2,4,4,1 mathematical method, the spectral differences were significantly enhanced with more defined absorption peaks, and the baseline shifts were also corrected. Figure 1 (c) shows the spectra treated with the 2,4,4,1 mathematical method combined with the SNVD scatter correction method, in which the spectral variation has been greatly reduced.

### Calibration and validation

The calibration and cross-validation statistics are shown in Table 2. Good NIRS prediction equations were obtained for both actually determined and calculated DE and ME, with relatively high RSQ_cal_ (0.86-0.89), R^2^_CV_ (0.84-0.87) and RPD_cv_ values (2.54-2.85) greater than 2.50. When applying these equations to validation samples, three of the four equations obtained good prediction performance with relatively high RSQ_v_ (0.84-0.86) and RPD_v_ values (2.53-2.69, >2.50).

**Table 2:** Calibration and cross-validation statistics of digestible energy (DE) and metabolizable energy (ME) of corn fed to growing pigs (MJ/kg, dry matter basis)

				Calibration					Cross-validation		
Items	Derivative	Scatter	Factors	*n*	Mean	SD^a^	SEC^b^	RSQ_cal_^c^	SECV^d^	R^2^_CV_^e^	RPD_cv_^f^
DE_D_^g^	2,12,12,1	SNVD	13	86	16.41	0.759	0.196	0.89	0.266	0.87	2.85
ME_D_^g^	2,10,10,1	SNVD	11	85	15.98	0.741	0.206	0.87	0.267	0.86	2.78
DE_C_^h^	2,10,10,1	SNVD	12	87	16.46	0.728	0.201	0.88	0.273	0.85	2.67
ME_C_^h^	2,8,8,1	SNVD	10	86	16.20	0.711	0.215	0.86	0.280	0.84	2.54

^a^Standard deviation

^b^Standard error of calibration

^c^Coefficient of determination for calibration

^d^Standard error of cross-validation

^e^Coefficient of determination for cross-validation

^f^RPD_CV_ = SD/SECV

^g^DE_D_ and ME_D_ values determined by digestion-metabolism experiments using growing pigs

^h^DE_C_ and ME_C_ values calculated according to the equations of Noblet and Perez [6]

**Fig. 2:**
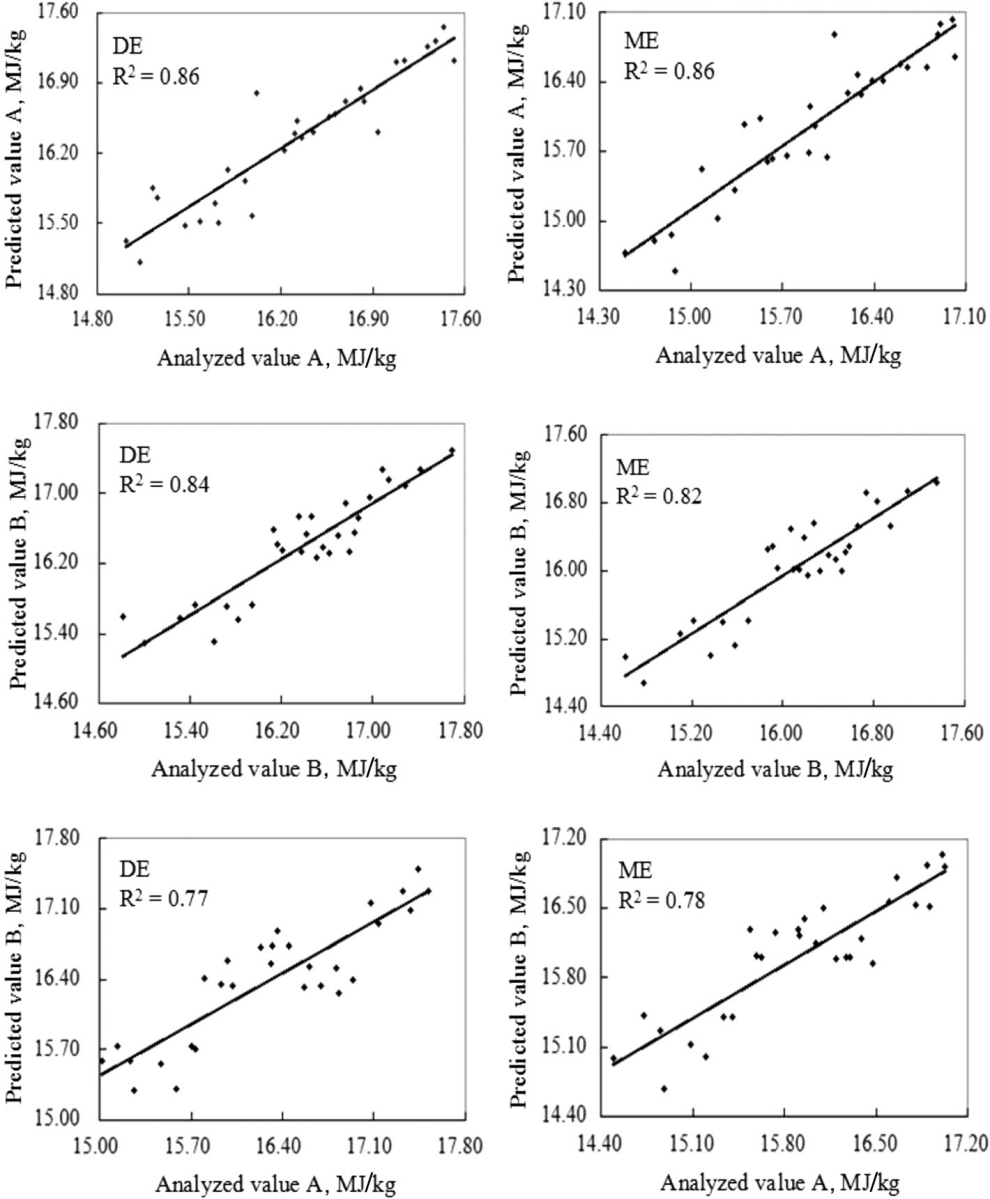
Plot of analyzed values versus NIRS predicted values of energy content of corn. Analyzed value A and analyzed value B were determined by digestion-metabolism experiments and the prediction equations of Noblet and Perez [6], respectively; Predicted value A and predicted value B were predicted by NIRS calibration equation built with energy values determined by digestion-metabolism experiments and the prediction equations of Noblet and Perez [6], respectively

Only the prediction equation for ME_C_ produced a relatively poor result with RSQ_v_ of 0.82 and RPD_v_ of 2.38 (<2.50) (Table 3). The relationship between analyzed values and NIRS predicted values for both actually determined and calculated DE and ME for the validation set are shown in Fig. 2. Except for ME_C_ (*R*^*2*^ = 0.82), the regression plots for DE_D_, ME_D_ and DE_C_ showed good performance with *R*^*2*^ all greater than 0.84. The results of linear regression analysis between actually DE_D_ values and values predicted by NIRS equations built from DE_C_ was not satisfactory with a relatively low *R*^*2*^ (0.77), and the same situation was found for ME (*R*^*2*^ = 0.78).

**Table 3:** Validation statistic of DE and ME of corn fed to growing pigs (MJ/kg, dry matter basis)

Constituent	*n*	Mean	SD^a^	SEP^b^	RSQ_v_^c^	RPD_v_^d^
DE_D_^e^	29	16.31	0.741	0.275	0.86	2.69
ME_D_^e^	29	15.93	0.730	0.276	0.86	2.64
DE_C_^f^	29	16.38	0.708	0.279	0.84	2.53
ME_C_^f^	29	16.10	0.676	0.284	0.82	2.38

^a^Standard deviation

^b^Standard error of prediction

^c^Coefficient of determination for validation

^d^RPD_v_ = SD/SEP

^e^DE and ME values determined by digestion-metabolism experiments using growing pigs

^f^DE and ME values calculated according to the equations of Noblet and Perez [6]

## Discussion

The average CP, EE, ash, ADF and NDF values of corn samples measured in this study were similar to that presented by NRC [2], but the variation in these components were larger than previous studies [3, 15, 32]. In this study, relatively precise DE_C_ and ME_C_ values were obtained due to the accurate results of the chemical analysis with low SEL and RSEL values (Table 1). It has practical application values to accurately predict the DE and ME content which could reduce the need for metabolism studies.

The average actual DE_D_ and ME_D_ values of the corn samples determined by digestion-metabolism experiments were similar with the values published by the NRC [2], while the DE_C_ and ME_C_ values were a little higher than the NRC [2] values. Due to the large variation in chemical composition of the collected corn samples, relatively wide ranges in corn DE and ME contents were obtained with relatively high coefficients of variation (CV all beyond 4.20 %, Table 1), which is essential for the development of robust prediction equations by NIRS [4, 5, 18]. With similar sample distributions, the calibration set and validation set samples were also suitable for the establishment of excellent NIR prediction equations [26].

Due to the relatively large number and precise reference data with large variation in DE and ME content of the corn samples used here, good calibration equations were obtained for both actually determined and calculated DE and ME, and except for the prediction equation built from ME_C_, good predictive performances were obtained for all the other three prediction equations which can be used for routine analysis. In previous studies, limited sample numbers and analysis error made it difficult to gain excellent NIRS performance for DE and ME content for pigs. Garnsworthy et al. [18] predicted the DE by NIRS using 33 wheat samples, but their results were not satisfying with a low coefficient of determination (*R*^*2*^ *=* 0.17). For a set of duplicate scans from 39 barley samples, a relatively poor result was also reported with a R^2^_CV_ of 0.69 for swine DE content [16]. Because of the inter-laboratory variation on the reference sample set, the performance of NIRS calibrations were also not good in the studies of [17] (R^2^_cal_ = 0.72, RPD_CV_ = 2.13) and [4] (R^2^_CV_ = 0.79). Although good calibration results for DE and ME (RSQ_cal_ = 0.87 and 0.86, respectively) were obtained in the study of Aufrère et al. [33], there was no data presented on validation statistics. It is probable that the relationship between actual and NIRS predicted values would be weakened in some validation exercises. Xiccato et al. [34] predicted the DE concentration of rabbit diets by NIRS and reported a high coefficient of determination (*R*^*2*^ *=* 0.90). However with validation, this relationship was weakened and the SECV increased. This result has also been reported by Deaville et al. [35] who predicted *in vivo* ME of whole crop cereals fed to sheep by NIRS and found that the *R*^*2*^ declined considerably between calibration (*R*^*2*^ *=* 0.87) and cross-validation process (R^2^_CV_ = 0.79, RPD_cv_ = 2.16). As a result, in order to decide whether the NIRS prediction performance is good or not, both results of calibration and validation should be taken into consideration.

In the present study, using the 2nd instead of 1st derivative gave better prediction performance, which is in agreement with previous studies [4, 5, 24, 36]. With the treatment by the 2nd derivative, the spectral differences were enhanced [37] and baseline shifts caused by sample particle size was removed [38]. Among different scatter correction methods used, the Standard Normal Variate plus Detrend correction (SNVD) gave the best results for both actually determined and predicted DE and ME.

Compared with the NIRS prediction equations built from DE_C_ or ME_C_ values, the prediction equation obtained from actually DE_D_ or ME_D_ values received a better predictive performance with higher RSQ_v_ and RPD_v_ values and lower SEP value. In addition, the relationship between DE_D_ or ME_D_ and values predicted by NIRS equations built from DE_C_ or ME_C_ was relatively poor. These results indicate that it is better to do the NIRS calibration for DE and ME based on reference data determined by digestion-metabolism experiments. The reason for this result might be that the prediction equations built by Noblet and Perez [6] were for complete feeds for pigs, when they were extrapolated to predict the DE and ME content of feed ingredients, the performance of them might be decreased because of the differences in chemical composition between complete feed and individual ingredients [39]. However, when time is limited and the conditions do not allow for digestion-metabolism experiments, the DE_C_ and ME_C_ values could also be used to do the NIRS calibrations with an acceptable performance [5]. In practice, the good NIRS prediction performance for DE and ME obtained in this study offered an advantage to effective use of corn resources in swine production.

## Conclusion

In summary, the results obtained in this study indicate that NIRS can be used as a routine analysis method for the rapid determination of DE and ME content in corn fed to growing pigs. Optimization of spectral pre-treatment methods can improve the NIRS calibration and prediction performance. NIRS prediction equations built from actual DE and ME content determined by digestion-metabolism experiments showed better prediction performance than those based on predicted DE and ME content calculated according to the equations of Noblet and Perez [6], but the latter could also be used for the NIRS calibration with an acceptable performance in some situations.

## Abbreviations

ADF, acid detergent fiber; CF, crude fiber; CP, crude protein; CV, coefficient of variation; DDGS, distillers dried grains with solubles; DE, digestible energy; DM, dry matter; EE, ether extract; GE, gross energy; IMSC, inverse multiplicative scatter correction; ME, metabolizable energy; MPLS, modified partial least squared; NDF, neutral detergent fiber; NIRS, near-infrared reflectance spectroscopy; R^2^_CV_, coefficient of determination for cross-validation; RPD, ratio of the standard error of performance to standard deviation; RSEL, relative standard error of laboratory; RSQ_cal_, coefficient of determination for calibration; RSQ_v_, coefficient of determination for validation; SD, sample standard deviation; SEC, standard error of calibration; SECV, standard error of cross-validation; SEL, standard error of laboratory; SEP, standard error of prediction; SMSC, standard multiplicative scatter correction; SNV, standard normal variate; SNVD, standard normal variate plus detrend correction; WMSC, weighted multiplicative scatter correction
