# Erratum to: Use of near-infrared reflectance spectroscopy for the rapid determination of the digestible energy and metabolizable energy content of corn fed to growing pigs

**DOI:** 10.1186/s40104-016-0108-6

**Published:** 2016-08-24

**Authors:** Juntao Li, Quanfeng Li, Defa Li, Yiqiang Chen, Xiaoxiao Wang, Wenjun Yang, Liying Zhang

**Affiliations:** State Key Lab of Animal Nutrition, College of Animal Science & Technology, China Agricultural University, Beijing, 100193 China

## Erratum

Unfortunately, the original version of this article [1] contained an error. The name of one of the authors was included incorrectly and it read “Wunjun Yang” instead of “Wenjun Yang”.

The name has been updated in the original article and is also correctly included in full in this erratum.
